# Dermoscopic Findings of Nevus of Ota

**DOI:** 10.4274/balkanmedj.galenos.2019.2019.11.46

**Published:** 2020-02-28

**Authors:** Ömer Faruk Elmas, Asuman Kilitçi

**Affiliations:** 1 Department of Dermatology, Kırşehir Ahi Evran University School of Medicine, Kırşehir, Turkey; 2 Department of Pathology, Kırşehir Ahi Evran University School of Medicine, Kırşehir, Turkey

To the Editor,

Nevus of Ota, also known as oculodermal melanocytosis, is characterized by a macular pigmentation usually localized on the forehead and periocular region. It typically shows a dermatomal distribution and the dermatomes of the first two branches of trigeminal nerve are typically involved (1,2). The diagnosis is usually based on the clinical appearance. In some cases, however, histopathological examination may be necessary. Dermoscopy is a non-invasive diagnostic tool enhancing the diagnostic accuracy in many dermatological diseases including both pigmented and non-pigmented ones. There is a comprehensive literature about the dermoscopic characteristics of many pigmented lesions, however, no data regarding the dermoscopic features of nevus of Ota exists in the relevant literature except for a single case report (3). Herein, we aimed to describe dermoscopic features of the lesions having a clinicopathological diagnosis of nevus of Ota.

Dermoscopic examination was performed by a polarized handheld dermoscope with x10 magnification (Dermlite 4, 3GEN Inc, San Juan Capistrano, CA, USA). The dermoscopic images were captured using a high-resolution mobile camera phone attached to the dermoscope (iPhone 7 plus, Apple Inc, CA, USA).

A total of 10 patients including 6 males and 4 females diagnosed with the nevus of Ota was enrolled. A written informed consent was obtained from all the participants. The mean age of the patients was 32 years (range 17-62). All of the lesions showed unilateral distribution. The most common localization was forehead (Figure 1a) followed by periorbital region. Scleral involvement was observed in 4 cases. All of the patients were consulted to the ophthalmologist and, none of the patients showed glaucoma or uveal melanoma. The most common dermoscopic findings were brown and gray structureless areas with a patchy distribution (Figure 1b, 1c) and scattered brown- gray dots (Figure 1b, 1c, 1d) which were observed in all of the lesions. Eight lesions showed terminal hairs (Figure 1d), and the fine scales were observed in 2 lesions. 4 lesions exhibited white clods in a “four dots clod” arrangement (Figure 1b, 1d). Perifollicular hypopigmentation was seen in 6 lesions (Figure 1d). Two lesions revealed yellowish structureless areas. As for vascular structures, serpentine vessels were observed in 2 lesions. Distribution of the dermoscopic findings was homogenous in all the lesions. Reticular pigmentation pattern was not detected in any case. Histopathologically, all of the lesions showed pigmented dendritic melanocytes and melanophages dissecting bundles of dermal collagen in reticular dermis (Figure 2a, 2b). The clinical, dermoscopic and histologic features of the patients are summarized in the Table 1.

Nevus of Ota is a type of dermal melanocytosis. Mongolian spot, nevus of Ito, nevus of Hori, and blue nevus are the other forms of dermal melanocytosis (1,2), and can usually be differentiated from nevus of Ota on the clinical grounds.

We described a peculiar dermoscopic pattern for nevus of Ota comprising gray structureless areas and scattered brown-gray dots. In the study of Zinoune et al. (3), dermoscopic features of a case of nevus of Ota was described as bluish to slate grey homogenous pigmentation. Exogenous ochronosis may present with a nevus of Ota-like clinical appearance showing speckled distribution of grayish brown macules. In a case study, dermoscopic features of exogenous ochronosis have been reported as “dark brown globules, elongated and curvilinear-worm like structures” (4). In the present study, none of the lesions showed a similar dermoscopic pattern. Lentigo maligna and lentigo maligna melanoma also enter into the differential diagnosis of nevus of Ota. Obliterated hair follicles, asymmetric pigmented follicular openings and pigmented rhomboidal structures are the strong dermoscopic clues to lentigo maligna and lentigo maligna melanoma (5,6).

The histopathological differential diagnosis of the nevus of Ota includes Mongolian spots and nevus of Ito which are the other main types of patchy dermal melanocytosis. The presence of the scattered dendritic melanocytes in dermis is a consant histopathological features for all these three entities. Lack of disturbance in dermal architecture and the presence of some stromal fibrotic reaction might be present both in the nevus of Ota and nevus of Ito. Lack of a stromal reaction and fibrosis may be a diagnostic clue to Mongolion spot. However, it is difficult to say that there is a distinct histological differentiation between these entities (7). In this context, a clinical and pathological correlation is essential for the diagnosis.

To the best of our knowledge, this is the first case series focusing on the dermoscopic features of nevus of Ota. Here we identified a peculiar dermoscopic pattern for the entity which may help the clinician to establish an accurate diagnosis.

## Figures and Tables

**Table 1:**
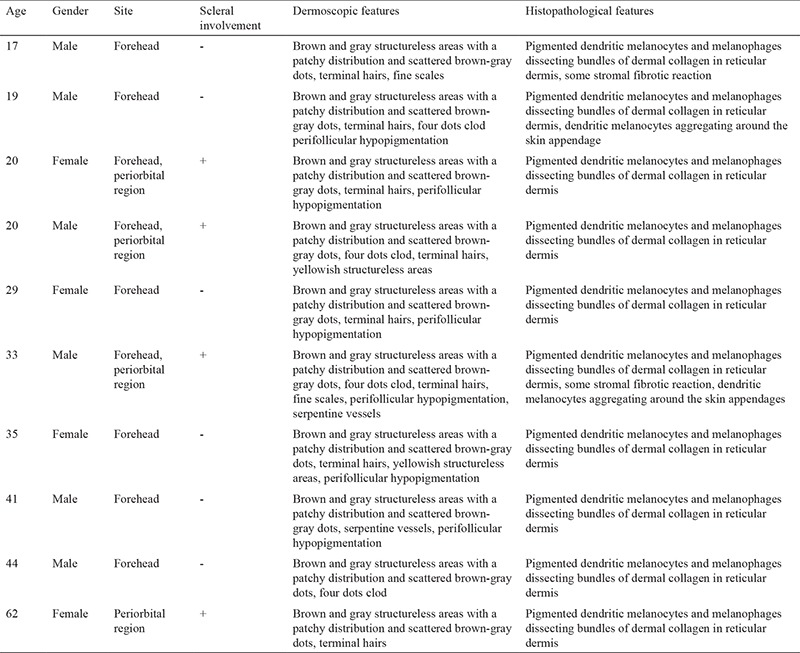
The clinical, dermoscopic and histopathological features of the patients with nevus of Ota

**Figure 1:**
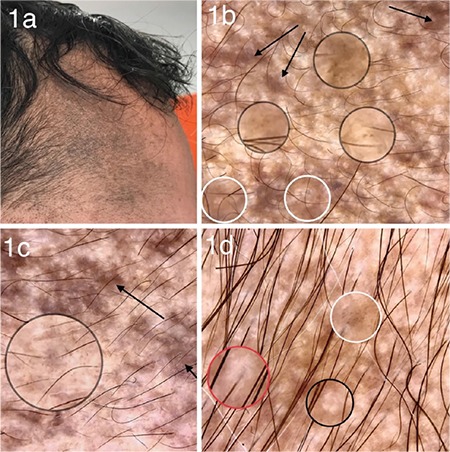
(a) Clinical appearance of nevus of Ota localized on the forehead of a young male patient. (b) Brown and grey structureless areas with patchy distribution (black arrows), scattered brown dots (grey circles). (c) Brown and grey structureless areas with patchy distribution (black arrows) and scattered brown dots (grey circle). (d) Scattered brown dots (white circles), four-dots-clod (black circle) and terminal hair with perifollicular hypopigmentation (red circle).

**Figure 2:**
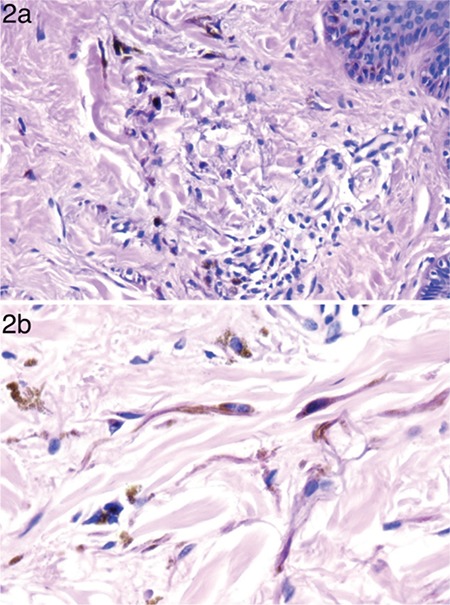
(a) Pigmented dendritic melanocytes in reticular dermis (x200, H&E), (b) Pigmented dendritic melanocytes and melanophages dissecting bundles of dermal collagen in reticular dermis (x400, H&E).

## References

[1] Dinulos JG (2015). Dermal melanocytosis and associated disorders. Curr Opin Pediatr.

[2] Harrison-Balestra C, Gugic D, Vincek V (2007). Clinically distinct form of acquired dermal melanocytosis with review of published work. J Dermatol.

[3] Zinoune S, Douhi Z, Baybay H, Elloudi S, Mernissi F-Z (2019). Bilateral Nevus of Ota: An Unusual Presentation. Madridge J Case Rep Stud.

[4] Khunger N, Kandhari R (2013). Dermoscopic criteria for differentiating exogenous ochronosis from melasma. Indian J Dermatol Venereol Leprol.

[5] Bollea-Garlatti LA, Galimberti GN, Galimberti RL (2016). Lentigo Maligna: Keys to Dermoscopic Diagnosis. Actas Dermosifiliogr.

[6] Micantonio T, Neri L, Longo C, Grassi S, Di Stefani A, Antonini A, et al (2018). A new dermoscopic algorithm for the differential diagnosis of facial lentigo maligna and pigmented actinic keratosis. Eur J Dermatol.

[7] Baykal C, Yılmaz Z, Sun GP, Büyükbabani N (2019). The spectrum of benign dermal dendritic melanocytic proliferations. J Eur Acad Dermatol Venereol.

